# Long COVID and Its Impact on Daily Functioning: Findings from the Si-Panda Behavioural Insights Survey in Slovenia

**DOI:** 10.2478/sjph-2026-0013

**Published:** 2026-06-01

**Authors:** Helena Jeriček Klanšček, Maruša Rehberger, Andreja Belščak Čolaković, Marina Šinko, Darja Lavtar, Ada Hočevar Grom

**Affiliations:** National Institute of Public Health, Trubarjeva cesta 2, 1000 Ljubljana, Slovenia

**Keywords:** SARS-CoV-2 virus, Consequences, Long COVID, Number of infections, Risk of a depressive disorder, SARS-CoV-2 virus, posledice, dolgi covid, število okužb, tveganje za depresijo

## Abstract

**Introduction:**

Research on the long-term consequences of COVID-19 has initially focused on the symptoms and prevalence of long COVID. However, few studies have fully incorporated the World Health Organization definition or explored its diverse predictors, including mental health factors. This study aims to deepen the understanding of long-term outcomes of COVID-19 and their associated factors.

**Methods:**

Data were drawn from the SI-PANDA Behavioural Insights survey on COVID-19, an online questionnaire administered to a selected sample of participants from an online access panel in Slovenia. The study included 5,961 participants aged 18 to 74. A multivariate logistic regression model was used to identify factors associated with reporting long COVID.

**Results:**

Among the 5,961 respondents, 3,234 reported having been infected with SARS-CoV-2 at least once. Of those, 38% reported persistent fatigue and lack of energy. Long COVID developed in 16.1% (n = 520) of respondents who had been infected. The factor most strongly associated with long COVID was experiencing at least one severe episode of COVID-19, which was associated with a fourfold increase in the odds (OR = 3.99; 95% CI: 3.25–4.91). Other significant associations were observed for risk of a depressive disorder (OR = 2.50; 95% CI: 1.79–3.44), three or more SARS-CoV-2 infections (OR = 2.30; 95% CI: 1.45–3.64), risky stress behaviour (OR = 2.10; 95% CI: 1.38–3.30), and the presence of at least one chronic disease (OR = 1.50; 95% CI: 1.24–1.91).

**Conclusions:**

Understanding and effectively addressing infectious diseases like COVID-19 requires not only insight into the virus’s biology and evolution but also recognition of the important role of mental health and psychological factors.

## Izvleček

**Uvod:**

Raziskave o dolgoročnih posledicah okužbe s covidom-19 so se sprva osredotočale predvsem na simptome in razširjenost dolgega covida. Vendar le malo študij vključuje definicijo dolgega covida, kakor ga definira Svetovna zdravstvena organizacija, ali raziskuje raznolike napovednike, vključno s kazalniki duševnega zdravja. Namen te študije je poglobiti razumevanje dolgoročnih izidov po covidu-19 in z njimi povezanih dejavnikov.

**Metode:**

Podatki so bili zbrani v spletni raziskavi SI-PANDA na vzorcu oseb, vključenih v komercialni spletni panel. V analizo je bilo vključenih 5961 odraslih, starih med 18 in 74 let. Za prepoznavanje dejavnikov, povezanih s poročanjem o dolgem covidu, smo uporabili multivariatni logistični regresijski model.

**Rezultati:**

Med 5961 anketiranci jih je 3234 poročalo, da so bili vsaj enkrat okuženi z virusom SARS-CoV-2. Od teh jih je 38 % poročalo o vztrajajoči utrujenosti in pomanjkanju energije. Dolgi covid se je razvil pri 16,1 % (n = 520) okuženih. Dejavnik, ki je bil najmočneje povezan z dolgim covidom, je bila izkušnja vsaj ene hude epizode covida-19, kar je bilo povezano s približno štirikrat večjimi obeti za pojav dolgega covida (OR = 3,99; 95 % IZ: 3,25–4,91). Poleg tega je bilo z dolgim covidom pomembno povezano tudi tveganje za depresivno motnjo (OR = 2,50; 95 % IZ: 1,79–3,44), tri ali več okužbe s SARS-CoV-2 (OR = 2,30; 95 % IZ: 1,45–3,64), tvegano stresno vedenje (OR = 2,10; 95 % IZ: 1,38–3,30) ter prisotnost vsaj ene kronične bolezni (OR = 1,50; 95 % IZ: 1,24–1,91).

**Zaključki:**

Učinkovito razumevanje in obravnavanje infekcijskih bolezni, kot je covid-19, zahteva ne le poznavanje biologije in evolucije virusa, ampak tudi prepoznavanje ključne vloge duševnega zdravja in psiholoških dejavnikov tveganja.

## 1 INTRODUCTION

The COVID-19 pandemic not only triggered a major health crisis but also exposed vulnerabilities in global systems and their limited preparedness for such extraordinary events (1).

Significant efforts to track and understand the biology and evolution of this pathogen in real time offer hope of anticipating its future evolutionary trajectories and developing prevention and treatment strategies (2,3,4). To support such advancements, there is a need to critically review not only the clinical course of infection but also to develop a broader understanding of the virus’s spread and its long-term consequences.

Research has documented connections between stress, mental health, and immune functioning, highlighting the role of psychological and behavioural factors in the course of viral infections (5,6,7). During the COVID-19 pandemic, levels of stress, anxiety, and related mental health challenges increased due to isolation, fear of infection, and economic uncertainty (8,9,10,11,12). In this context, behavioural insights and mental health indicators are particularly relevant, as they may influence both vulnerability to infection and recovery processes.

One of the most important long-term consequences of SARS-CoV-2 infection is the so-called “post-acute COVID syndrome” or simply “long COVID” (13). The World Health Organization (WHO) defines it as a condition occurring in individuals with a history of probable or confirmed infection with the SARS-CoV-2 virus, appearing 2–3 months after the onset of the disease, with symptoms lasting at least 2 months that cannot be explained by an alternative diagnosis and substantially affecting an individual’s daily functioning (14, 15). Symptoms may appear after initial recovery from an acute episode of COVID-19, persist from the onset of the illness, and fluctuate or recur over time (16). International data show a wide variation in prevalence estimates, ranging from 0% to 93% in a global systematic review (17). Research on the long-term consequences of COVID-19 initially focused on the symptoms and prevalence of long COVID, and its associations with socio-demographic characteristics and other diseases.

Although the WHO definition of long COVID includes not only symptom duration but also its impact on daily functioning, this aspect is not always consistently operationalised or explicitly examined in empirical studies (16, 17). Many studies primarily focus on the presence and persistence of symptoms, while the extent to which these symptoms affect everyday life—such as work, employment, and family functioning—is less frequently explored.

Therefore, it is important to consider not only whether long-term symptoms are present, but also the degree to which they impact daily functioning, as this represents an important dimension of the overall burden of long COVID.

However, it has been found that women, the elderly, those with chronic diseases, and individuals with lower socioeconomic status (SES) are more likely to experience or exhibit more symptoms of long COVID (17,18,19,20,21,22). Some studies also emphasised the importance of stress and mental health issues (23). However, relatively few studies fully consider the WHO definition and examine the various associations with long COVID, including factors related to COVID-19 disease severity, chronic conditions, and mental health.

The objective of this paper is to deepen understanding of the long-term consequences of COVID-19 infection, taking into account not only long-term symptoms but also their impact on daily life. We explore associations with factors such as the number and severity of infections, vaccination, chronic diseases, risky stress behaviour, and the risk of a depressive disorder.

## 2 METHODS

### 2.1 Survey design and data collection

The source of data was the SI-PANDA Behavioural Insights survey on COVID-19, an online questionnaire administered to a selected sample of participants from an online access panel in Slovenia. All research participants were voluntary members of the commercial online access panel JazVem/Opinia.Club. The source population comprised all panel members aged 18–74 years. Recruitment was conducted using stratified quota sampling by gender, age, and statistical region. Informed consent was obtained electronically before participation. Participants confirmed their voluntary involvement by selecting the option “I agree to participate in the research” after confirming they were at least 18 years old and had read the research information. Those who selected “I do not agree to participate in the research” were excluded from the research. Approximately 1,000 adults aged 18–74 participated in each of the 7 rounds of the survey, conducted monthly between September 2022 and March 2023 (data collection in each round lasted 4 days). The response rate varied from 19% in the last two rounds to 30% in the first round of data collection. Up to 3 participations per respondent were allowed across 7 rounds of the survey, but this analysis included only unique units. We included only the most recent participation to ensure the reported data reflected the most recent available information (n = 5,961). The median interview length ranged from 10.3 minutes in the second round to 15.5 minutes in the 7th round. Responses to all questions were mandatory. The survey data were weighted by gender, age group, and statistical region to ensure representativeness. The research data supporting the findings of this study are openly available in the Zenodo repository (https://doi.org/10.5281/zenodo.18479765) (24). The study protocol was reviewed and approved by the National Medical Ethics Committee of the Republic of Slovenia (KME RS) in September 2022.

### 2.2 Variable and indicator definitions

Age was grouped into four categories: 18–29, 30–49, 50–64, and 65–74 years. Education levels were consolidated into two categories: secondary or less and tertiary or higher. Employment status was categorised as active (employed) and non-active (students, retired, unemployed).

Explanatory variables included reported SARS-CoV-2 vaccination status (yes/no) and the reported presence of at least one chronic disease (diabetes, asthma, hypertension or other cardiovascular diseases, chronic obstructive pulmonary disease or other pulmonary diseases, chronic headache including migraines).

Risky stress behaviour was operationalised as a binary variable (1 = yes; 0 = no), indicating the co-occurrence of frequent stress and difficulties in coping. This variable was based on items derived from the Finbalt Health Monitor methodology (25) and has been used in the CINDI Health Behaviour Surveys in Slovenia (NIJZ) since 2001. Participants were classified as exhibiting risky stress behaviour (1 = yes) if they met both of the following criteria: reporting frequent stress, defined as selecting “Often” or “Every day” in response to the question: “How often did you feel tense, stressed, or under a lot of pressure in the last 14 days?”, and reporting poor coping, defined as selecting “I still have a lot of trouble coping with them” or “I can’t handle them; my life is almost unbearable” in response to the question: “How do you manage the tensions, stress and pressures you experience in life?” All other participants were classified as not exhibiting risky stress behaviour (0 = no).

Mental well-being was assessed using the WHO-5 questionnaire. The answers to the 5 survey questions were recoded. Then the sum of all answers of the recoded variable was divided into three categories: 0–28 “risk of a depressive disorder”, 29–50 “poor mental well-being” and 51–100 “excellent mental well-being”.

Exploratory indicators included the number of self-reported SARS-CoV-2 infections (1, 2, 3 or more) and whether any infection was severe.

Participants reported specific symptoms at 3 months after any infection with SARS-CoV-2, persisting for at least 2 months after recovering from SARS-CoV-2: fatigue, reduced physical capacity, muscle and joint pain, headache, cough, problems with concentration and/or memory, sleep disturbances, problems with the perception of taste and/or smell, chest pain, shortness of breath, unpleasant feelings of fear, sadness, anger, tearfulness, negative thoughts, heart palpitations, rhythm disturbances, digestive problems, menstrual problems, or other. They also noted how these symptoms affected their ability to work, manage household tasks, or care for others (not at all, a little, a lot, or extremely).

### 2.3 Long COVID definition

The question we used to define the symptoms and their duration was: “Three months after you recovered from SARS-CoV-2 infection, did you have (or do you still have) any of the following problems that lasted (last) for at least 2 months and that you did not have before the infection?” To measure how the reported long COVID symptoms affect work and home life, we used the question ‘How much did these issues impact the following areas?’, focusing on respondents who answered “a lot” or “extremely”. Long COVID was operationalised as a binary variable (1 for long COVID, 0 for no long COVID). Participants were classified as 1 (long COVID) if they reported at least one symptom occurring 3 months after any prior SARS-CoV-2 infection, with a duration of at least 2 months, that had a very strong or extreme impact on their work or home life. All other participants were classified as 0 (no long COVID).

### 2.4 Data analysis

Data analysis commenced with the assessment of the significance of multiple comparisons between the non-infected group and individuals who had been infected with SARS-CoV-2 at least once, using the Bonferroni correction test, with a significance threshold set at p < 0.05. A similar analytical approach was subsequently applied to the infected cohort (respondents who reported being infected with SARS-CoV-2 at least once) to explore potential differences associated with long COVID. A multivariate logistic regression model was employed to examine associations with the development of Long COVID, incorporating nine imputed variables derived from a comprehensive literature review and prior analyses. Odds ratios (ORs) and their corresponding 95% confidence intervals (CIs) were estimated for each variable. All logistic regression assumptions were met, with multicollinearity and model stability within acceptable limits. The reference group for comparison was selected based on the lowest incidence of long COVID. Statistical significance was defined as a two-sided p-value < 0.05. Data cleaning and analysis were performed using IBM SPSS version 25.

## 3 RESULTS

**Figure 1.**
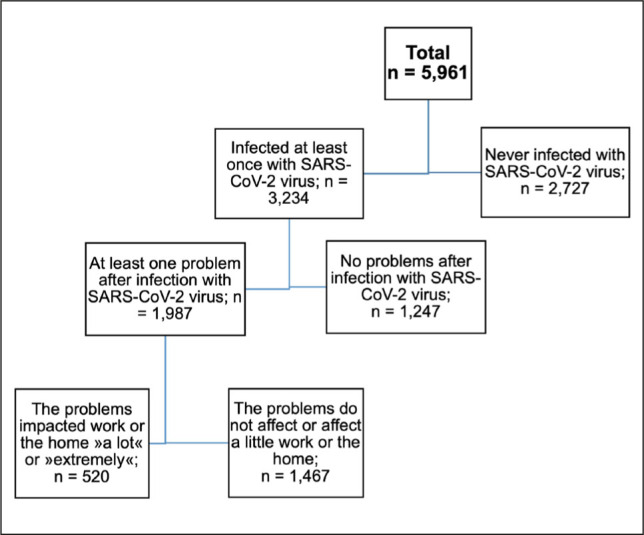
Structure of the studied sample.

Out of 5,961 respondents, 3,234 had been infected with the SARS-CoV-2 virus at least once. Among these, 1,987 respondents experienced at least one symptom lasting at least 2 months and not present before the infection. After recovering from COVID-19, 16.1% (n = 520) of infected respondents reported that they had at least one symptom, which, according to their assessment, impacted their work/job or their ability to manage their home, family, or household “a lot” or “extremely” (Figure 1). According to the WHO definition, these respondents developed long COVID.

Characteristics of respondents who have been infected at least once with SARS-CoV-2 vs those who have never been infected are presented in Table 1.

**Table 1.** Characteristics of respondents who have never infected vs. those infected at least once with the SARS-CoV-2.

		**Never infected**	**Infected with SARS-CoV-2 at least once**	
	
		**% (n)**	**% (n)**	**p**
**Total**	Total, 18–74 years	45.8 (2,727)	54.2 (3,234)	
**Gender**	Male	48.2 (1,314)	46.6 (1,507)	0.222
	Female	51.8 (1,413)	53.4 (1,727)	
**Age**	18–29	11.4 (310)	18.5 (598)*	< 0.0001
	30–49	33.1 (902)	45.3 (1,466)*	
	50–64	33.1 (902)*	25.2 (814)	
	65–74	22.5 (613)*	11.0 (356)	
**Education**	Secondary or less	57.6 (1,572)*	47.1 (1,525)	< 0.0001
	Tertiary or higher	42.4 (1,156)	52.9 (1,709)*	
**Employment status**	Active	55.3 (1,507)	69.1 (2,233)*	< 0.0001
	Non-active	39.5 (1,076)*	26.8 (868)	
**Vaccinated against SARS-CoV-2**	No	22.5 (613)	33.2 (1,074)*	< 0.0001
	Yes	77.5 (2,114)*	66.8 (2,160)	
**At least one chronic disease**	No	61.1 (1,668)	63.9 (2,065)*	0.013
	Yes	38.9 (1,060)*	36.1 (1,169)	
**Risky stress behaviour**	No	96.1 (2,621)	96.1 (3,109)	0.910
	Yes	3.9 (107)	3.9 (125)	
**Mental well-being**	Risk of a depressive disorder	8.4 (230)	9.2 (297)	< 0.0001
	Poor mental well-being	19.4 (529)	24.5 (792)*	
	Excellent mental well-being	72.2 (1,969)*	66.3 (2,145)	

Legend:

* p value < 0.05 (statistically significant difference between respondents infected with SARS-CoV-2 at least once and those never infected; the * indicates a significantly larger percentage in the category of the explanatory variable).

**Figure 2.**
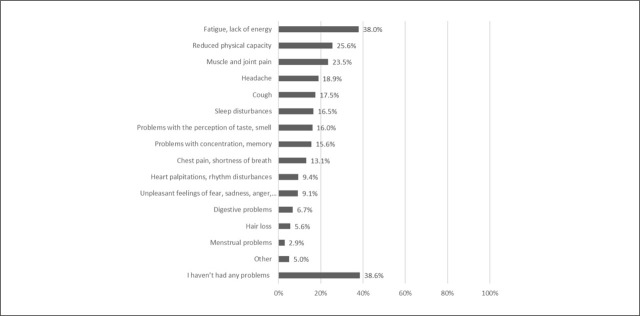
Presence of persistent problems 3 months after any infection with SARS-CoV-2 that lasted for at least 2 months.

Statistically significant differences between never-infected and at least once-infected respondents were found in age, education, employment status, vaccination against SARSCoV-2, the presence of at least one chronic disease, and well-being. At least once-infected respondents were more often younger, more educated, active (employed), without chronic diseases, and with poorer mental well-being (Table 1). Among all at least once-infected respondents, 68.3% had been infected once, and 3.5% had been infected three times or more. For 25% of respondents, the course of at least one infection was severe, while for 75% it was asymptomatic or mild. 38.6% reported no problems after any infection with SARS-CoV-2, 13.2% reported one persistent problem, 12% reported two problems, 11% reported three problems, and 25.2% reported four or more persistent problems at least 2 months after the infection (Figure 2).

Statistically significant differences between at least once-infected respondents who developed long COVID and those who did not were found in all variables listed in Table 2, except for vaccination against SARS-CoV-2, education, and employment status. Among the respondents who developed long COVID, there were more women, those with risky stress behaviour, a risk of a depressive disorder or poor well-being, multiple infections, at least one severe course of illness, and the presence of at least one chronic disease (Table 2).

**Table 2.** Characteristics of respondents who have been infected at least once with SARS-CoV-2, with and without long COVID (n = 3,234).

		**Without Long COVID**	**With Long COVID**	
	
		**% (n)**	**% (n)**	**p**
**Total infected**	Total infected, 18–74 years	83.9 (2,714)	16.1 (520)	
**Gender**	Male	47.8 (1,297)*	40.4 (210)	0.002
	Female	52.2 (1,417)	59.6 (310)*	
**Age**	18–29	18.5 (503)	18.2 (94)	0.009
	30–49	45.0 (1,221)	47.0 (245)	
	50–64	24.7 (669)	27.8 (145)	
	65–74	11.8 (320)*	7.0 (36)	
**Vaccinated against SARS-Cov-2**	No	33.7 (915)	30.5 (159)	0.154
	Yes	66.3 (1,798)	69.5 (362)	
**Education**	Secondary or less	46.9 (1,273)	48.3 (251)	0.573
	Tertiary or higher	53.1 (1,440)	51.7 (269)	
**Employment status**	Active	69.0 (1,872)	69.4 (361)	0.849
	Non-active	31.0 (841)	30.6 (159)	
**At least one chronic disease**	No	66.0 (1,792)*	52.6 (274)	< 0.0001
	Yes	34.0 (922)	47.4 (247)*	
**Risky stress behaviour**	No	97.4 (2,644)*	89.3 (464)	< 0.0001
	Yes	2.6 (69)	10.7 (56)*	
**Mental well-being**	Risk of a depressive disorder	7.3 (197)	19.2 (100)*	< 0.0001
	Poor mental well-being	23.1 (627)	31.6 (164)*	
	Excellent mental well-being	69.6 (1,889)*	49.2 (256)	
**Infected with SARS-CoV-2**	Once	70.3 (1,907)*	58.4 (304)	< 0.0001
	Twice	26.9 (729)	34.7 (180)*	
	Three times or more	2.9 (78)	6.9 (36)*	
**At least one severe episode of COVID-19**	No	80.5 (2,185)*	46.1 (240)	< 0.0001
	Yes	19.5 (528)	53.9 (280)*	

Legend:

* p value < 0.05 (statistically significant difference between respondents without long COVID and those with long COVID; the * indicates a significantly larger percentage in the category of the explanatory variable).

Table 3 shows the results of the multivariate logistic regression analysis, identifying factors associated with long COVID (n = 3,234; p < 0.001; explained variance = 18.9%) among respondents who were infected at least once with SARS-CoV-2. Factors that were statistically significantly associated with the occurrence of long COVID included age, risky stress behaviour, mental well-being, number of infections, vaccination status, illness severity, and the presence of at least one chronic disease. Gender, education, and employment status were not statistically significantly associated with long COVID. Respondents aged 50–64 had the highest likelihood of developing long COVID. People engaging in risky stress behaviour had twice the odds of developing long COVID compared to those who do not. People at risk of a depressive disorder were most likely to develop long COVID. The number of SARS-CoV-2 infections is also associated with the risk of long COVID, with those infected 3 or more times having the highest risk. Vaccinated individuals also have a higher risk of developing long COVID than those who were not vaccinated, did not intend to be vaccinated, or could not be vaccinated for health reasons. People who experienced at least one severe episode of COVID-19 have four times higher odds of developing long COVID compared to those who had a mild or asymptomatic episode. Additionally, people with at least one chronic disease have higher odds of developing long COVID than those without (Table 3).

**Table 3.** Logistic regression results of factors associated with the occurrence of long COVID.

							**95% C.I. for EXP(B)**	
						
	**B**	**S.E.**	**Wald**	**df**	**Sig.**	**Exp(B)**	**Lower**	**Upper**
**Age (65–74)**	**Reference**							
(18–29)	0.441	0.234	3.558	1	0.059	1.554	0.983	2.456
(30–49)	0.626	0.237	6.977	1	0.008	1.870	1.175	2.975
(50–64)	0.608	0.230	6.993	1	0.008	1.837	1.170	2.883
**Gender (Male)**	**Reference**							
(Female)	0.184	0.107	2.972	1	0.085	1.202	0.975	1.481
**Education (Tertiary or higher)**	**Reference**							
(Secondary or less)	0.064	0.107	0.352	1	0.553	1.066	0.864	1.315
**Employment status (Non-active)**	**Reference**							
(Active)	−0.024	0.142	0.028	1	0.866	0.976	0.739	1.289
**At least one chronic disease (No)**	**Reference**							
(Yes)	0.431	0.109	15.596	1	< 0.0001	1.538	1.242	1.905
**Vaccinated against SARS-CoV-2 (No)**	**Reference**							
(Yes)	0.278	0.115	5.843	1	0.016	1.321	1.054	1.655
**Risky stress behaviour (No)**	**Reference**							
(Yes)	0.759	0.222	11.657	1	0.001	2.136	1.382	3.302
**Mental well-being(Excellent mental well-being)**	**Reference**							
(Risk of a depressive disorder)	0.908	0.167	29.672	1	< 0.0001	2.478	1.788	3.435
(Poor mental well-being)	0.502	0.118	18.015	1	< 0.0001	1.652	1.310	2.083
**Infected with SARS-CoV-2 (Once)**	**Reference**							
(Twice)	0.254	0.115	4.906	1	0.027	1.290	1.030	1.615
(Three times or more)	0.830	0.236	12.397	1	< 0.0001	2.293	1.445	3.640
**At least one severe episode of COVID-19 (No)**	**Reference**							
(Yes)	1.385	0.106	171.414	1	< 0.0001	3.993	3.246	4.913
Constant	−3.555	0.250	202.880	1	< 0.0001	0.029		

Legend: B – coefficients; S.E. – standard error; Wald – Wald statistic; df – degrees of freedom; Sig. – p - value; Exp(B) – odds ratio; C.I. – confidence interval

## 4 DISCUSSION

By March 2023, 54.2% of respondents reported SARS-CoV-2 infection, with the majority (68.3%) reporting a single infection. For most respondents (75%), the course of illness was asymptomatic or mild, while 25% experienced a severe episode. Despite this, 61.4% reported at least one persistent symptom lasting more than 2 months post-infection, and 25.2% reported four or more such symptoms. The most common symptoms were fatigue and lack of energy, followed by reduced physical capacity and muscle and joint pain. These findings are consistent with previous research. A scoping review of 34 studies with follow-up periods of up to 3 months post-COVID-19 reported that the most frequently observed physical health problems were fatigue (28%–87%), pain (myalgia 4.5%–36%), arthralgia (6%–27%), and reduced physical capacity (26). Additionally, a systematic review and meta-analysis of 61 cohort studies from 15 countries identified a very wide range of physical and psychological symptoms reported by individuals at least 12 weeks after SARS-CoV-2 infection (27).

Moreover, 16.1% of all infected respondents (n = 520) reported that these symptoms impacted their ability to work and manage their home, family, or household responsibilities “a lot” or “extremely”, indicating long COVID. This definition of long COVID is consistent with those used in other studies, though variations in methodology, including the timeframe for assessing symptom persistence and the type of population studied, limit direct comparability. Other studies assessed different symptoms without necessarily examining their impact on daily functioning. Additionally, different timeframes were used: we considered symptom persistence at 3 months, while other studies, for example, assessed symptoms after 4 weeks (26, 28). Moreover, variation in reported prevalence is also linked to the timing of the study—earlier or later in the pandemic—and to whether symptoms were measured in hospitalised or non-hospitalised patients. Mahoney (29) reports in a meta-analysis that the pooled prevalence of COVID-19 survivors experiencing at least one persistent symptom, regardless of hospitalisation status, was 45%.

Respondents infected with SARS-CoV-2 at least once were more likely to be aged 30–49, vaccinated, without chronic diseases, and with poorer mental well-being compared with those who were never infected. Vaccinated individuals and those without chronic diseases may have been less concerned about contracting the virus and therefore taken fewer precautions. Conversely, respondents with poorer mental well-being may have been less vigilant in adhering to preventive measures. Previous research supports the association between mental health and adherence to preventive measures (30, 31).

The findings suggest that the most significant factors associated with long COVID include age, risky stress behaviour, overall well-being, the number of infections, vaccination status, infection severity, and the presence of at least one chronic condition. Of these, 4 emerged as the most significant factors, with 2 related to infection characteristics: infection severity and having 3 or more infections. The other 2 factors are linked to mental health. Infection severity was identified as the strongest factor, increasing the odds of long COVID fourfold. This is consistent with findings in other studies, which also highlight the importance of infection severity as a key factor, as well as the impact of multiple infections (32,33,34). The mechanisms underlying these associations are not yet fully understood. Multiple infections likely contribute to prolonged inflammation and viral persistence, which may lead to additional harm or tissue damage across multiple organs and systems (23).

Interestingly, the second most significant factor associated with long COVID was poorer mental well-being, specifically the risk of a depressive disorder, a finding also reported in systematic reviews and meta-analyses (35). Additionally, risky stress behaviour was identified as a significant factor, which has also been shown to be important in several studies (36, 37). Kostev (38) proposes several possible hypotheses. The first suggests that mental health conditions are more strongly associated with the development of neuropsychiatric sequelae of COVID-19 than in the general population. He also notes that dysregulation of the hypothalamic-pituitary-adrenal axis is common in people with psychiatric disorders, and that these dysregulations may also play a role in the pathophysiology of post-COVID-19 condition.

The findings on vaccination are not fully aligned with most studies that underscore the effectiveness of COVID-19 vaccines in mitigating the incidence and severity of long COVID, particularly when administered before infection. In this study, more than half of the participants were unvaccinated. Furthermore, detailed data on vaccination timing and the specific COVID-19 variants involved were unavailable, which is a major limitation that may affect the interpretation of findings on vaccination effectiveness. It is plausible that some participants received vaccination only after contracting COVID-19, which may have influenced the observed outcomes. Previous research indicates that vaccination before infection substantially reduces the likelihood of long COVID. In contrast, post-infection vaccination provides limited benefit (39,40,41,42). The effectiveness of vaccination may also depend on variant-specific characteristics, as newer variants such as Omicron exhibit distinct immune-evasion profiles (42). Variations in study designs—such as meta-analyses integrating heterogeneous datasets versus cohort studies targeting specific populations—can also yield different conclusions. Furthermore, other studies have reported that certain chronic conditions, such as headaches or migraines, might emerge or worsen following COVID-19 (43,44,45). For instance, a cross-sectional study explored the impact of COVID-19 on pre-existing headaches and found that both de novo headaches and exacerbations of migraines were prevalent in post-COVID people (46).

### 4.1 Limitations

The study has several limitations that should be considered when interpreting the findings. First, the cross-sectional design limits causal inference and does not allow conclusions about the directionality of the observed associations. In particular, the temporal ambiguity of mental health measures means that these factors may represent either antecedents or consequences of long COVID. Second, the survey is subject to selection and reporting biases. All data were collected via a survey questionnaire; therefore, all variables—including infection status, vaccination status, and chronic conditions—are reported and may be affected by reporting inaccuracies. In addition, recall bias may be present, particularly regarding the timing and number of SARS-CoV-2 infections.

Participation in the online panel was voluntary and required internet access and digital literacy, which may limit representativeness. Response rates may have been lower among less-educated individuals, as online surveys tend to attract a more highly educated population. However, analyses by educational attainment take into account that younger participants may still be in education. Importantly, previous comparative analyses have shown strong agreement between reported COVID-19 vaccination data and data from the National Electronic Registry of Vaccinated Individuals and Adverse Events Following Vaccination, supporting the survey’s suitability for its intended purpose.

## 5 CONCLUSION

The findings of this study indicate that understanding and addressing long COVID requires a multidimensional approach that extends beyond biological factors to include mental health and behavioural dimensions. From a public health perspective, these results suggest that strategies targeting infectious diseases should integrate mental health promotion and stress management as core components, rather than treating them as separate domains. Interventions aimed at reducing psychological distress and strengthening coping capacity may not only improve well-being but also lead to more favourable recovery trajectories following infection.

The findings suggest that routine screening for mental health and stress-related factors may be beneficial among individuals with a history of COVID-19, particularly those at higher risk of prolonged symptoms. Incorporating brief, validated tools into primary care and post-COVID follow-up could facilitate the early identification of individuals who may benefit from timely psychosocial support or multidisciplinary care. Finally, future research should prioritise longitudinal designs to better disentangle causal relationships among mental health, behavioural factors, and long COVID. Further studies should also evaluate the effectiveness of integrated interventions addressing both physical and mental health, as well as the influence of socioeconomic and contextual factors.
